# Towards microvascular pressure estimation using ultrasound and photoacoustic imaging

**DOI:** 10.1016/j.pacs.2019.04.001

**Published:** 2019-05-30

**Authors:** Min Choi, Roger Zemp

**Affiliations:** Department of Electrical and Computer Engineering, Faculty of Engineering, University of Alberta, 9107 – 116 Street, Edmonton, AB, T6G 2V4, Canada

**Keywords:** Photoacoustic imaging, Ultrasound imaging, Perfusion, Hemodynamics

## Abstract

Microvascular pressure drives perfusion in tissues but is difficult to measure. A method is proposed here to estimate relative pressures in microvessels using photoacoustic and ultrasound tracking of small vessels during calibrated tissue compression. A photoacoustic–ultrasound dual imaging transducer is used to directly compress on tissue *in vivo*. Photoacoustic signals from blood vessels diminish as an external load is applied and eventually reaches a minimum or vanishes when external pressure is sufficiently greater than the internal pressure. Two methods were proposed to estimate relative pressures. In the first approach, vessels were tracked during compression and when the vessel photoacoustic signals vanished below a set threshold, the internal pressures were assigned as the external loading pressure at the respective collapse point. In this approach pressures required to collapse vessel signatures completely were found to be much greater than physiological blood pressures. An alternative approach was to track the cross-sectional area of small vessels with changing external load and fitting the data to a known Shapiro model for thin-walled vessel compression. This approach produced estimates of internal pressures which were much more realistic. Both approaches produced the same rank-ordering of relative pressures of various vessels *in vivo*. Approaches thus far require future work to become fully quantitative but the present contributions represent steps towards this goal.

## 1 Introduction

Microcirculation is a clinically important factor in determining many disease conditions including septic-shock, diabetes, hypertension, ischemia, cancer, and organ failure [1], [2], [3], [4]. Various imaging techniques, both invasive and non-invasive, such as orthogonal polarization spectral imaging (OPS), phase contrast magnetic resonance imaging (MRI), intravital microscopy (IVM), capillary microscopy, optical coherence tomography (OCT), ultrasound biomicroscopy, and photoacoustic microscopy have become important tools in understanding blood flow in small vessels [4], [5], [6], [7]. These approaches, however, provide little information about pressures in microvessels as downstream vascular resistance is typically unknown. Yet, pressure in vascular networks is a key parameter to driving perfusion and drug delivery efficacy. Few methods exist for estimating blood pressures in microvascular networks [8], [9].

Sphygmomanometry is the classic non-invasive technique used to measure blood pressure, but only for measuring systemic arterial blood pressure in the radial or brachial arteries, or sometimes in the femoral artery. Automated oscillometry methods perform similar tasks in these large arteries. Non-invasive methods for local blood pressure measurements include applanation tonometry with strain gage measurements or similar methods [10], [11]. While applanation tonometry is widely used to measure arterial pressure by applying external pressure using the tonometer tip, it cannot be resolved spatially so the pressure of vessels cannot be measure simultaneously [12]. Invasive catheters are commonly used for assessing blood pressures in large vessels or heart chambers. Compression ultrasound has additionally been used to estimate blood pressures in brachial arteries [13]. However, none of these approaches are suitable for estimating blood pressures in small vessels.

Being able to estimate the blood pressure for vessels of interest are important in areas such as surgery as surgeons needs to strategically decide which vessels to cut in order to reduce unnecessary hemorrhaging and minimize physiological change during the surgery [14], [15].

We propose new approaches to estimating relative pressures in small vessels using photoacoustic and ultrasound methods for tracking microvessels during tissue compression. High-frequency photoacoustic imaging offers the ability to image small vessels with high contrast and with robustness to tissue motion unlike Doppler ultrasound where the signals from tissue motion would cloud signals from vessel flow. Recently photoacoustic (PA) imaging has been combined with US imaging for visualizing microcirculation [16], [17], [18], [19], [20], [21], [22].

We introduce a compression-tracking photoacoustic–ultrasound dual imaging system based on a high-frequency 40 MHz ultrasound transducer. This system tracks the loading force temporally registered with ultrasound and photoacoustic image sequences. Using this method, we track the decreasing lumen area (as measured by photoacoustic imaging) with increasing applied external pressure. As the transmural pressure (the difference between the internal and the external pressure) decreases with greater external loading, the photoacoustic signal is seen to diminish as the interrogated vessel is pressed to buckle then collapse. Currently, this method cannot be used as a regular cuffing method because of our transducer shapes, however cuffing could be considered in future designs.

One method to estimate microvascular pressure involves tracking vessels during compression and when the vessel photoacoustic signals vanishes below a set threshold, the internal pressures are assigned as the external loading pressure at the respective collapse point.

A second approach used model fitting of the collapse process. Vessel cross-sectional areas as a function of compression force were fit to a model proposed by Shapiro et al., which was previously validated for thin-walled vessels [23]. The internal pressure was a model parameter estimated from the fitting procedure. Validation of proposed techniques in phantoms and in vivo are discussed, along with current limitations and future prospects.

## 2 Methods

### 2.1 Imaging system

A VEVO 2100 along with VEVO LAZR (Fujifilm, Visualsonics Inc.) are used to obtain both photoacoustic and ultrasound images simultaneously and in real-time. The LZ 550 transducer, which has a transmit frequency of 40 MHz, a receive bandwidth of 32–55 MHz and the capability to image up to 15 mm deep, has been used for all experiments. The wavelength of the excitation light is fixed at 805 nm, which is the isosbestic point, where the absorption coefficient of the oxy- and deoxy-hemoglobin are the same. Because of this property, the amount of generated acoustic signal from the illumination would not be biased towards either oxy- or deoxy-hemoglobin. The power of the laser is around 16 mJ/cm^2^ and the frame rate is 10 Hz.

**Fig. 1:**
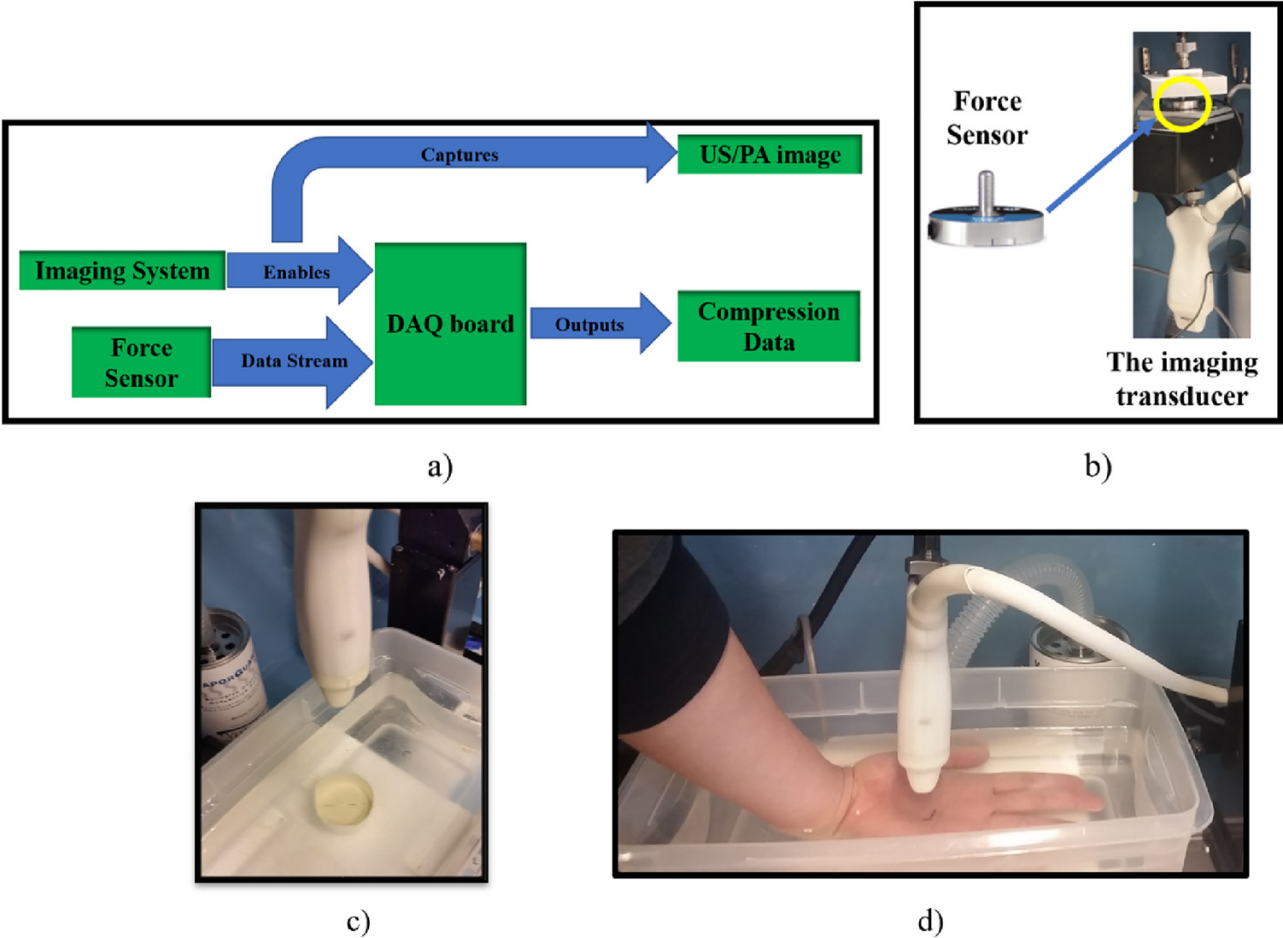
Overview of experimental setup: (a) schematic of image acquisitions synchronized with external loading force measurements, (b) the placement of the force sensor in the imaging transducer, the sample would be located directly under the transducer, (c) the phantom experiment setup and (d) the human subject experiment setup.

A force sensor (iload mini™, Loadstar Sensors Inc.), is placed between the transducer and a transducer holder with custom designed parts, as shown in Fig. 1. The force sensor can measure both compressive and tensile force up to 50 lb. The force sensor is connected to a signal converter which then continuously streams analog signal to the DAQ board (USB-1608GX, Measurement Computing Corporation ©). The DAQ board can be directly connected to the computer to log voltage amplitude, which is linearly related to the force being applied to the source. To synchronize the image capturing sequence and the applied force, the VEVO LAZR system is connected to the DAQ board where the VEVO LAZR system sends a signal when PA image sequence starts which, in turn, starts sending the force data to the computer. Because the trigger pulse is short, DAQ board is must be set to receive at least 100,000 samples per second. Additionally, a piece of gelatin is inserted into the transducer shell to provide flat and smooth interface between tissue layer and the transducer. This will prevent two pinch points from occurring when a vessel collapses. A manual translation stage is used to lower the transducer onto the imaging subject. In case of *in vivo* imaging, the compression process is kept in the order of multiple seconds in order to avoid the effects of viscoelastic relaxation.

### 2.2 Phantom experiments

For phantom experiments, swine blood is used. A phantom is created by placing a microcellulose tube (200 μ m diameter and 7 μ m wall thickness, #16706869, Leica Microsystems, Germany) in a 60 mm diameter Petri dish. Then, a gelatin solution (10% m/V) is poured to create ∼5 mm thick layer, effectively placing the tube in the middle of the phantom. One end of the tube is connected to a syringe. The other end of the tube is left open to let the liquid exit. A syringe pump is used with flow rates from 0.20 ml/min to 0.45 ml/min. Because of the small tube diameter, the 40MHz LZ 550 transducer is used to obtain images. Compression and imaging experiments were performed to validate that vessel collapse points increased with increasing internal pressure from increased flow rates.

### 2.3 Human subject experiments

For *in vivo* experiments, an arm and a hand of a human subject is used. A small area on the lower arm or on the hand will is pressed by the transducer while acquiring photoacoustic, ultrasound, and force data. The two methods for relative pressure estimation are used with the hypothesis that relative pressure in vessels would be estimated with similar rank-order between the methods. In such experiments, vessels at similar depths beneath the tissue surface were selected to minimize any depth-dependence of internally applied strains.

Human subject experiments were conducted in accordance with ethical protocols approved by the University of Alberta Health Research Ethics Board (Pro00007759).

### 2.4 Photoacoustic vessel-collapse estimation of relative pressures using vessel tracking

**Fig. 2:**
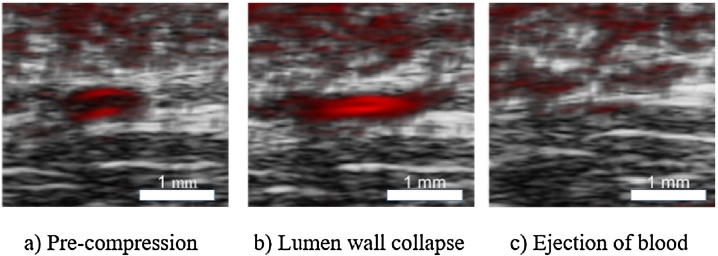
Collapse of a vessel tracked by photoacoustic–ultrasound dual imaging.

When a vessel is compressed, it will buckle and collapse, leading to loss of photoacoustic signal. When this signal drops below a 50% threshold we call this the effective collapse pressure. This pressure may not be the same as the internal pressure in the vessel, but may have diagnostic value as they may be related by some scaling factor and offset [24]. Fig. 2 shows ultrasound–photoacoustic images of the vessel collapse process.

To estimate the effective collapse pressures, we implemented a vessel tracking algorithm to identify the applied force at which each vessel vanishes within a video sequence. For each frame in the set, first thresholding is applied to suppress noise, small and irrelevant photoacoustic signals and all other signals outside the area of interest in order to visualize vessels of interest more clearly. This thresholding process is only applied once per frame. A region of interest is manually drawn around several vessels of interest. In the future this process could be automated. On the first frame, locations of the centroid of each vessel are determined and vessels are indexed. Then the next frame is loaded and the locations of the centroids in the next frame are determined. The distances between the each of the old centroids with all of the new centroids are calculated and the index number of the vessel is then assigned to the new vessel with the closest difference. The chosen centroid is then removed from the calculation process, which prevents vessel index numbers from overlapping. This method continues until the vessel is small enough so that the algorithm can no longer detect it.

After the entire frame has been processed, the first frame, with vessels color-coded based on the collapse pressure, is shown.

### 2.5 Model-based microvascular pressure estimation

Shapiro [23] formulated a phenomenological model describing the relationship between the area of blood vessels and an external pressure. The original equation by Shapiro is shown below:

(1) $$-\frac{p_{i}-p_{e}}{\kappa_{p}}\propto {\left(\frac{A}{A_{0}}\right)}^{-n}$$

where, *p*_*i*_ is blood pressure of the vessel, *p*_*e*_ is external pressure applied to the vessel, *κ*_*p*_ is critical pressure of vessel collapse, *A* is current cross-sectional lumen area, *A*_0_ is cross-sectional lumen area in the unstressed state and *n* is size reduction constant, known to be $\frac{3}{2}$ for veins [23].

Our strategy for measuring intravascular pressure is to track vessel area (as measured with photoacoustic imaging and ultrasound imaging) as a function of external loading and fit resulting data to the Shapiro model to extract internal pressure as a best-fit parameter. To do this, we perform the following steps: (1) manually select a few vessels and (2) apply an external force to compresses the tissue using the photoacoustic transducer head. (3) Track the effective vessel area (from the photoacoustic signal) as a function of applied compression. Here vessel areas are quantified using 50% photoacoustic thresholding, which is different from the first thresholding process. (4) Fit the vessel-area versus external pressure data to the above pressure-area equation to extract fitting parameters of vessel compliance and internal pressures.

## 3 Results

### 3.1 Phantom experiments

**Fig. 3:**
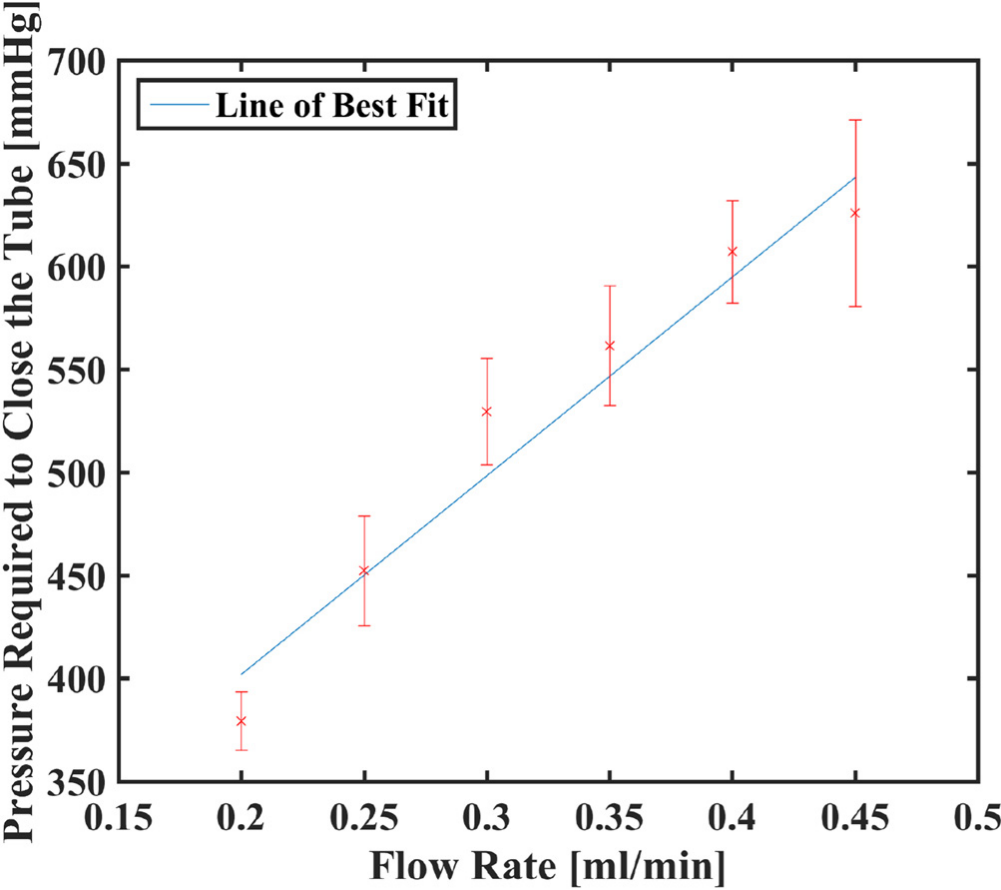
External pressure required to collapse cellulose tubes, four compressions were performed for each flow rate.

To demonstrate that the photoacoustic vessel-collapse estimation method produce relative pressure estimates which scale linearly with internal vessel pressures, we performed phantom experiments as described in Section 2. Four compressions were done for each flow rate and the load pressures applied by the transducer (as measured by the applied force divided by the transducer area) required to close the tube are 379 ± 14 mmHg, 452 ± 27 mmHg, 529 ± 26 mmHg, 561 ± 29 mmHg, 607 ± 25 mmHg and 626 ± 45 mmHg, for flow speeds of 0.2, 0.25, 0.3, 0.35, 0.4 and 0.45 ml/min, respectively. Therefore the percentage standard deviations are 3.72%, 5.89%, 4.89%, 5.18%, 4.09% and 7.24%. The thickness of gelatin phantoms decreased about 1–1.5 mm when it became no longer suitable for experimentation due to cracking. As shown in Fig. 3, the data set follows a linear trend, corresponding to Darcys Law. Compressions were performed within ∼1 s to minimize pressure buildup at the syringe pump and thus more closely achieve a constant input pressure. As the flow rate is decreased further, the pressures required to close the tube remained relatively constant. This is because the layer of gelatin becomes the dominant factor in determining the external force required to close the tube when the flow rate is below 0.20 ml/min. The tube was found to be too small (comparable with system resolution) to accurately use area-tracking and model fitting and the size of photoacoustic signature remained relatively similar throughout the compression until it vanished at some specific flow-rate-dependent external loading. Therefore, the phantom experiment was only used to validate relative pressures using the photoacoustic vessel-collapse estimation.

### 3.2 *In vivo* experiments

To demonstrate the ability of the photoacoustic vessel-collapse method to estimate relative pressures in human subjects we produced relative pressure maps of select vessels in the hand of a human subject. For simplicity, the estimated relative blood pressure in each vessel is shown on a percent scale, relative to the maximum applied external pressure. In addition to centroid tracking, the applied pressure at each frame is displayed along with tracked images to view the progression of compression more precisely. The external pressure measured using the force sensor for the compression shown in video 1 ranges from 0 mmHg to 1758 mmHg, which is beyond physiological blood pressure levels. The normalized pressures of vessels, which are measured collapse pressure divided by the maximum applied pressure, are 9%, 99.6% and 86.9% respectively.

To illustrate the model-fitting methods we tracked three vessels in the wrist of a human subject during transducer compressions. Vessels significantly larger than the system resolution were chosen for area tracking. The estimated values using the model-based estimation method is shown in Table 1. The goodness of fit for each vessel is shown in Fig. 6. While the model does not necessarily provide a strong fit to the data, realistic and quantitative parameter estimates were obtained.

**Table 1:** The estimated *κ*_*p*_ and *p*_*i*_ corresponding to Fig. 5.

Vessel #	*p*_*i*_ (mmHg)	*κ*_*p*_ (mmHg)
1	41.75	7.152
2	43.55	6.895
3	56.16	5.586

**Fig. 4:**
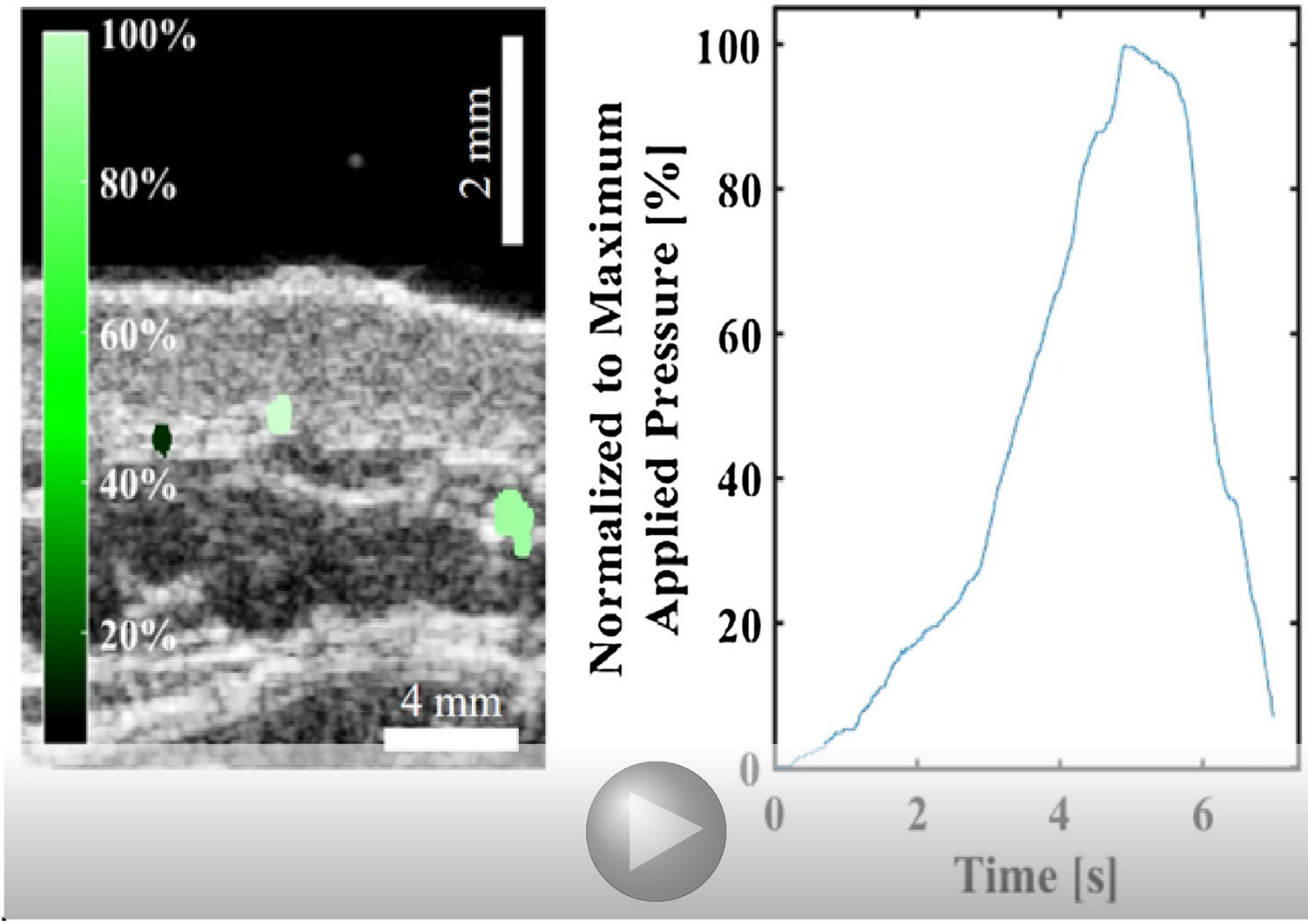
Tracking of vessel collapse due to external pressure and the estimation of relative blood pressure of tracked vessels located on the left arm of a human subject.

**Fig. 5:**
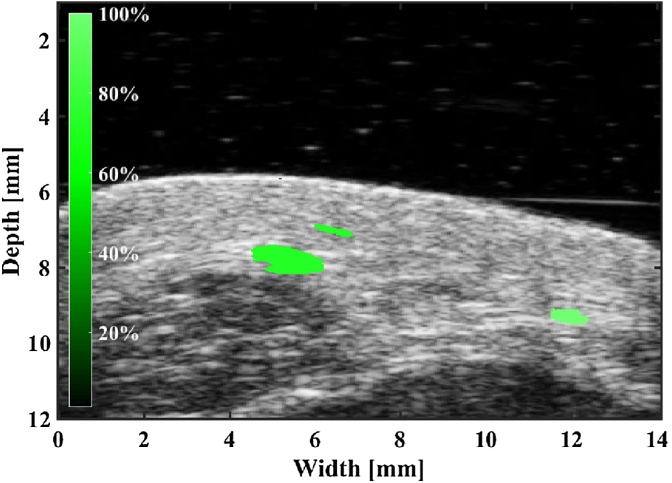
Estimation of relative blood pressures of the tracked vessel located near the left wrist of a human subject using the same tracking method used in Fig. 4.

**Fig. 6:**
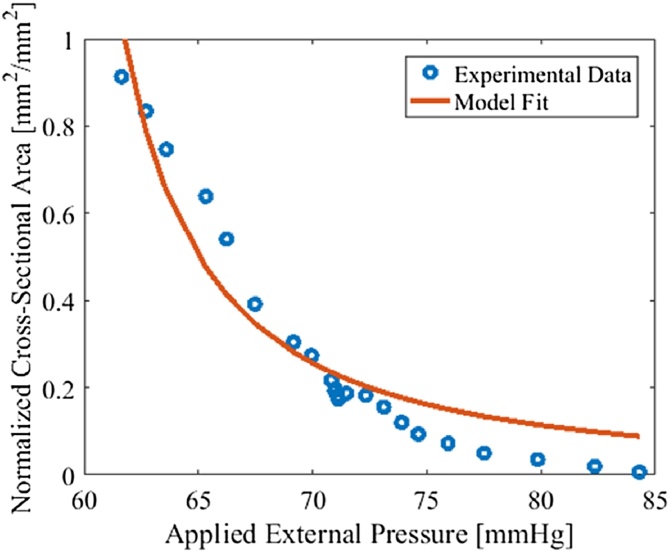
An example of data fitting using Shapiro's equation on the tracked vessels shown in Fig. 5.

We additionally used the same dataset for comparison of estimation methods. The maximum applied external pressure required to collapse the vessels was 89 mmHg and the estimated normalized pressures of vessels are 59.8%, 60.3% and 82.8% from left to right. Note that the relative pressures in vessels 1 and 2 appeared similar in both methods. Moreover, the relative pressure in vessel 3 was significantly greater than in vessels 1 and 2 as estimated with both methods.

To demonstrate the repeatability of the relative pressure estimation methods, single vessels were tracked over multiple compression cycles. Table 2 shows sample results from these experiments, demonstrating repeatability of pressure estimation with a standard deviation of 7.8 mmHg. Similar repeatability was seen with the vessel-collapse method.

**Table 2:** The estimated *κ*_*p*_, *p*_*i*_ and the external loading pressure for each compression for standalone model-based estimation method on a single vessel.

Compression #	*p*_*i*_ (mmHg)	*κ*_*p*_ (mmHg)	External loading *p* (mmHg)
1	33.02	6.783	81.70
2	29.16	7.175	72.39
3	27.33	10.82	78.98
4	32.06	11.14	93.89

## 4 Discussion

This experiment is among first attempts at estimating microvascular pressures non-invasively. For vessel-collapse method, a naive hypothesis was that vessels collapse (and lead to disappearance of photoacoustic signals) when external pressures overcome internal pressures. In actuality, more detailed analysis of the literature reveals that the vessel collapse process is a complex one involving buckling and pinching, but that much larger external pressures are required to collapse the vessel than exist internally [23], [25], [26].

For the phantom experiment, small tubes with thin walls are selected because we are interested specifically in estimating pressure of small vessels in the body. Also, while the outline of the tube could not be detected with B-mode ultrasound, the blood within the tube could still be imaged with photoacoustic imaging, and the vanishing of the photoacoustic at some externally applied pressure could be observed.

Compression *in vivo* is largely effected by viscoelastic relaxation, which can hinder accurate measurement of pressure. This effect is non-linear and is dependent on the speed and the magnitude of the compression as well as composition of the tissue layer. There are various reports in regards to the viscoelastic time constants of soft tissue but they range from 0.2 s and 3 s to over 70 s depending on the speed of stress application [27], [28], [29]. Our strategy was to apply compression on a time scale of a few seconds aiming to be faster than the viscoelastic relaxation rate. However this rate is unknown and dependent on the rate of compression requiring future analysis.

Besides unknown viscoelastic tissue properties other challenges exist for quantitative pressure estimation using the vessel-collapse method. For example, a small compression area is known to lead to systematic overestimation as it is with pressure cuffs. Because of this, external loading much higher than physiological pressure is required to collapse the vessel fully, which the simple vessel tracking method estimates as the internal pressure. In contrast to the first method, the model fitting method predicts the internal pressure of the vessel by assuming how the vessel shape changes as the external pressure is varied. This method is considered more accurate as it is based on phenomenological observation of isolated thin-walled vessels and it accounts for various mechanical factors of a vessel. Quantification challenges could be in part remedied in future work by measuring blood pressure in major vessels for calibration.

For the model-based pressure estimation method, the Shapiro equation is purely phenomenological and designed for isolated thin-walled vessels. Current model fitting has some discrepancies, which may in part due to deficiencies of the Shapiro model and future work should seek for improved models. The degree of the quality of fit on different vessels may vary depending on the location of the vessel in terms of the loading direction and with respect to the surrounding environment. Because both photoacoustic and ultrasound images are a slice of a 3D volume, maintaining the direction of the vessel compression and tissue movement parallel to the direction of the external pressure is crucial. In addition, the mechanical properties of various vessel types will play a significant role in how area changes with external force. For arteries, they will not collapse even without blood flow as arterial walls support them from closing. However, veins and capillaries do not have such support and will collapse when flow stops. On top of that, flows in the arteries and arterioles are more pulsatile than veins and capillaries. While the pulsing of the artery can be seen, it is harder to measure blood pressure of the arteries using transducer compression with continuously changing area. Veins, on the other hand, will provide more accurate pressure values as it does not vary greatly with each pulse. Furthermore, it is impossible to measure the pressure in the capillaries as it is difficult to distinguish them from noise due to resolution of the transducer. However, when compressed, small signals in the image vanish and the change in the background PA signal may indicate density of capillaries within the imaging region.

In both methods, vessel-area tracking is limited to vessels larger than the system resolution and the variation between patients cannot be accounted for as the estimated pressure is purely relative to each other within each imaging set. However, vessel-collapse method can be used to track cluster of vessels with sizes near the system resolution in order to estimate perfusion by determining how quickly the photoacoustic signal diminishes [30]. So, while limitations to both methods cause quantitative microvascular pressure estimation to be difficult, relative pressure estimation methods may provide a means of assessing perfusion irregularities and curves of photoacoustic signal versus loading could prove to have diagnostic significance even if measures are not yet fully quantitative. To achieve improved quantification, additional work is needed, including modeling tissue stiffness, modeling internal pressure re-distribution, modeling vessel collapse and vessel wall biomechanics, among other complexities. Present work has focused on the loss of signal during compression due to exclusion of blood volume. Release of tissue compression may also have significant value for quantifying re-fill rates, as we recently demonstrated.

## 5 Conclusions

We have demonstrated an ability to use US-PA dual imaging to measure pressures of microvessels locally and relative to each other. This is the first attempt at directly and non-invasively quantifying microvascular pressure (*in vivo*). The sizes of vessels were around 200 μ m to 1 mm, which correspond to larger arterioles and venules. Also, the measured pressure values are much greater than the expected physiological values and may differ significantly for each compression due to the visco-elastic relaxation of soft tissues. The pressure tracking can rank local blood vessels in terms of the applied pressure, which can reveal circulation path, potential clots, microvascular resistance and perfusion status. If calibration techniques are successfully developed, the actual pressure inside each vessel can be calculated, which could be used to measure blood pressure in venules and capillaries within relatively short time frame.

### Conflicts of interest

The authors have no relevant financial interests in this article and no potential conflicts of interest to disclose. R. Zemp is Co-Founder of illumiSonics Inc., which, however, did not support this work.

### Funding

We gratefully acknowledge funding from NSERC (355544-2008, 375340-2009, STPGP 396444), Terry-Fox Foundation and the Canadian Cancer Society (TFF 019237, TFF 019240, CCS 2011-700718), Alberta Innovates Health Solutions AIHS CRIO Team Award 201201154, the Alberta Cancer Research Institute (ACB 23728), the Canada Foundation for Innovation, Leaders Opportunity Fund (18472), Alberta Advanced Education and Technology, Small Equipment Grants Program (URSI09007SEG), and Alberta Ingenuity/Alberta Innovates scholarships for graduate and undergraduate students.
